# Child Abuse Consultation Rates Before vs During the COVID-19 Pandemic in Japan

**DOI:** 10.1001/jamanetworkopen.2023.1878

**Published:** 2023-03-09

**Authors:** Xerxes Seposo, Aden Kay Celis-Seposo, Kayo Ueda

**Affiliations:** 1 Department of Hygiene, Faculty of Medicine, Graduate School of Medicine, Hokkaido University, Sapporo, Japan; 2 School of Tropical Medicine and Global Health, Nagasaki University, Nagasaki, Japan

## Abstract

This cross-sectional study examines the association between the first 2 years of the COVID-19 pandemic and child abuse consultations in 47 Japanese prefectures.

## Introduction

Globally, the COVID-19 pandemic has had varying implications for various health outcomes and age groups.^1^ The pandemic has affected children and adolescents immensely, particularly due to restrictions on society, which may be associated with widening educational disparities, worsening child mental health issues, and environments inducing maltreatment.^2^ Evidence on the consequences of pandemic-related restrictions, however, has been scarce in the Asian region, wherein these restrictions are still enforced. In this study, we examined the association between the pandemic and the number of child abuse consultations in Japan.

## Methods

Child abuse was defined as any of the following acts being committed by a caretaker (parent or guardian) on a person younger than 18 years: physical abuse, neglect, sexual abuse, and psychological abuse.^3^ Details on the reporting of abuse under the Japanese system are given in eMethods in Supplement 1. Hokkaido University deemed this cross-sectional study exempt from ethics review and waived the informed consent requirement because publicly available aggregated and anonymized data were used. We followed the STROBE reporting guideline.

We obtained the publicly available 2019 to 2021 monthly number of child abuse consultations and estimated the child abuse consultation rates in 47 prefectures from the Ministry of Health, Labour and Welfare in Japan.^3^ The pandemic binary term was encoded with 0 for 2019 (prepandemic period) and 1 for 2020 to 2021 (pandemic period). We used an interrupted time series method to estimate the association of the pandemic with consultation rates for the first-stage, prefecture-level analysis. Subsequently, the first-stage analysis estimates were pooled using a random-effects meta-analysis model to generate the nationwide association. Parameterization of methods is described in the eMethods in Supplement 1. Analyses were performed using R, version 4.2.0 (R Core Team).

## Results

**Figure 1.**
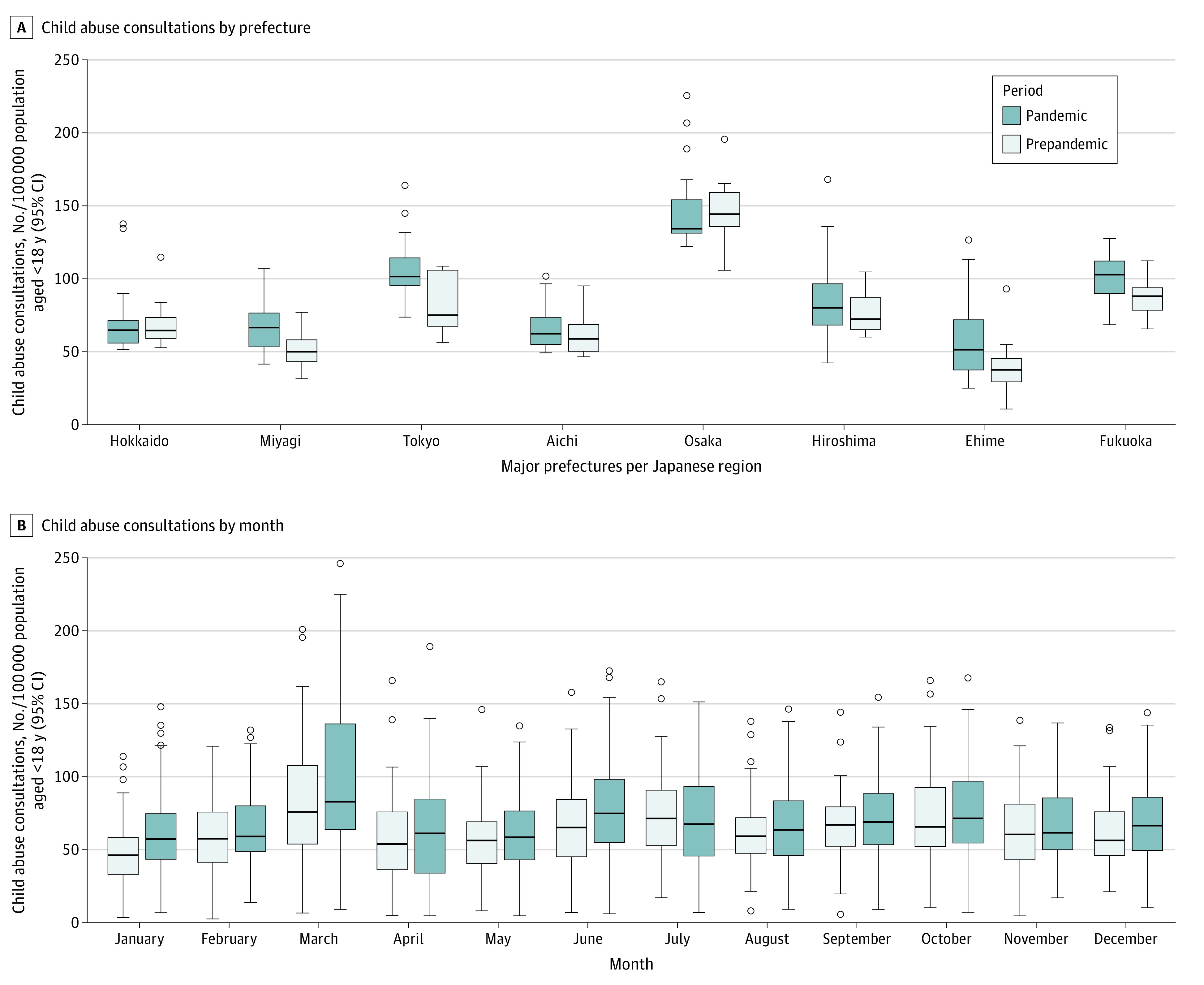
Prefecture-Specific and Month-Specific Child Abuse Consultation Rates The error bars represent the 95% CIs. Circles indicate observations; horizontal lines in the boxes, median values; bottom edges of the boxes, minimum values; top edges of the boxes, maximum values.

**Figure 2.**
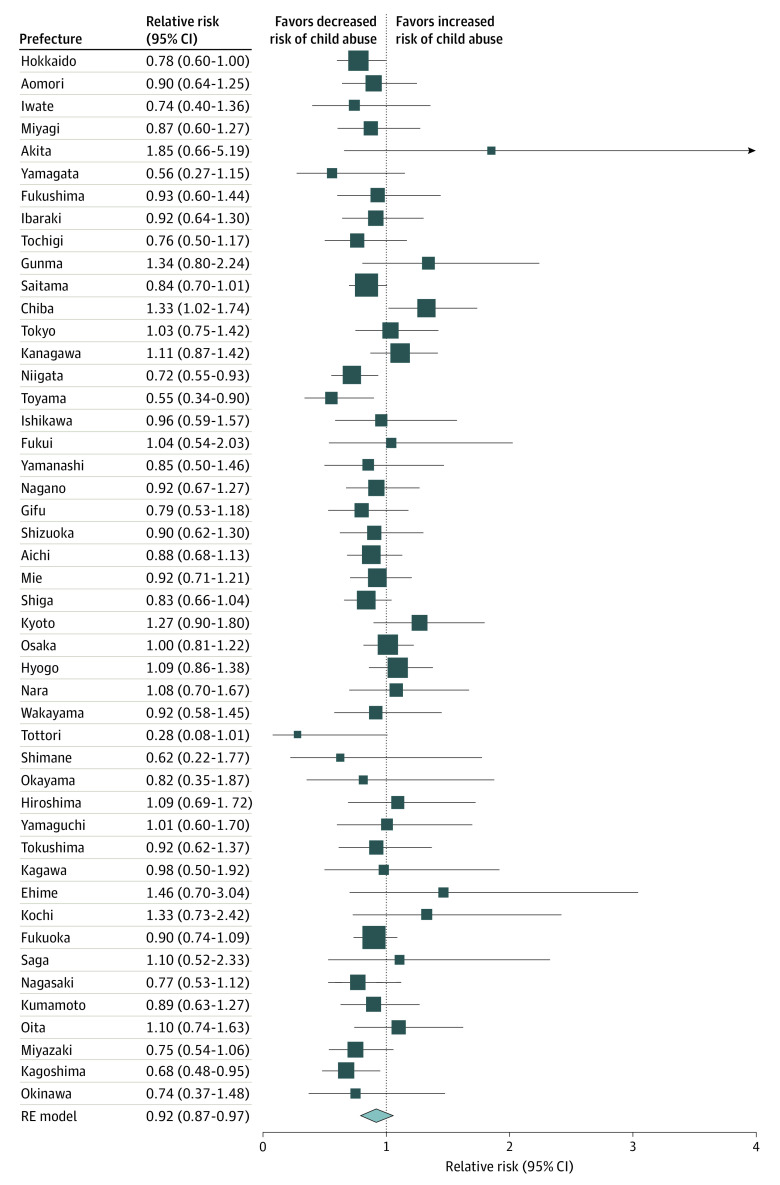
Prefecture-Specific and Nationwide Risk of Child Abuse The graph shows the risk of child abuse consultation during the pandemic compared with before the pandemic. The size of the box represents the amount of uncertainty for each of the central estimates of each prefecture. The lowest estimate is either the pooled or nationwide estimate. The vertical dotted line represents the null association. Error bars represent 95% CIs. RE indicates random effects.

From 2019 to 2021, on average, 182 549 child abuse consultations were recorded per year in Japan. The highest median (IQR) consultation rate was in Osaka (pandemic: 134.85 [132.06-154.53] per 100 000 population; prepandemic: 144.89 [136.60-159.46] per 100 000 population), and the lowest was in Tottori (pandemic: 8.97 [7.57-13.73] per 100 000 population; prepandemic: 7.29 [4.48-11.77] per 100 000 population). We did not observe any consistent pattern in median consultation rates between periods; however, the highest consultation rate was during March (pandemic: 82.74 [63.70-136.04] per 100 000 population; prepandemic: 75.86 [54.08-107.67] per 100 000 population) (Figure 1). Consultation rates decreased statistically during the pandemic, with nationwide risks reduced by 8.32% (95% CI, −13.32% to −3.02%) compared with the prepandemic risks (Figure 2).

## Discussion

The overall reduction in consultation rates during the pandemic was similar to that in the study by Swedo et al,^4^ who noted a lower number of emergency department visits during the pandemic. This finding is further supported by Sege and Stephens,^5^ who noted that the nonincrease in child abuse proportions may be associated with family support systems, which helped buffer financial distress and household difficulties, thereby preventing maltreatment. Several local studies found that favorable parenting behaviors and open stream parental communication were more frequent during the pandemic.^6^

The study has 2 key limitations. First, child abuse consultation was used as a proxy for actual medical-confirmed maltreatment. Further studies are warranted to determine the extent to which consultations can be a proxy for actual maltreatment cases. Second, the results reflected the local settings; thus, caution is warranted when interpreting these results in other settings. In this study, we found reduced child abuse consultation rates during the first 2 years of the COVID-19 pandemic in Japan.

## Notes

Supplement 1.
eMethods
**eFigure.** Child Abuse Response and Assessment Flowchart
eReferences

Supplement 2.
Data Sharing Statement
