# Supplementary material for: Child Abuse Consultation Rates Before vs During the COVID-19 Pandemic in Japan
Source: JAMA Netw Open. 2023 Mar 9;6(3):e231878. doi: 10.1001/jamanetworkopen.2023.1878 (PMC9999241; doi:10.1001/jamanetworkopen.2023.1878)
Supplement: Supplement 1. — eMethods eFigure. Child Abuse Response and Assessment Flowchart eReferences [file jamanetwopen-e231878-s001.pdf]

Supplementary Online Content

Seposo X, Celis-Seposo AK, Ueda K. Child abuse consultation rates before vs during the COVID-19 pandemic in Japan. *JAMA Netw Open*. 2023;6(3):e231878. doi:10.1001/jamanetworkopen.2023.1878

eMethods.

eFigure. Child Abuse Response and Assessment Flowchart

eReferences

This supplementary material has been provided by the authors to give readers additional information about their work.

© 2023 Seposo X et al. *JAMA Network Open.*

eMethods.

Child abuse and child abuse reporting system

The definition of a child under Japanese laws is those who are 18 and younger. As prescribed by the laws preventing child abuse, the national and local governments develop systems that intend to prevent child abuse. The relevant child abuse consultations in this study are reported by Child Guidance Centers, which are administrative agencies established under the Child Welfare Law. The Child Guidance Centers accept consultations and notifications regarding children under 18 from anyone, including children themselves, their families, schoolteachers, and residents. The Child Guidance Center is a specialized counseling agency that helps families think together and solve problems so that all children can grow up physically and mentally healthy and maximize their potential.[1] The child abuse consultations at first instance, are not medical in nature, since these are based on the professionally trained Child Guidance center staff as shown in eFigure 1. In certain circumstances, however, an investigation by the Child Guidance staff is hampered due to the lack of other information; apart from location, the public health centers or relevant health centers will be involved, thus making the specific child abuse consultation medical in nature.

© 2023 Seposo X et al. *JAMA Network Open.*

eFigure. Child Abuse Response and Assessment Flowchart

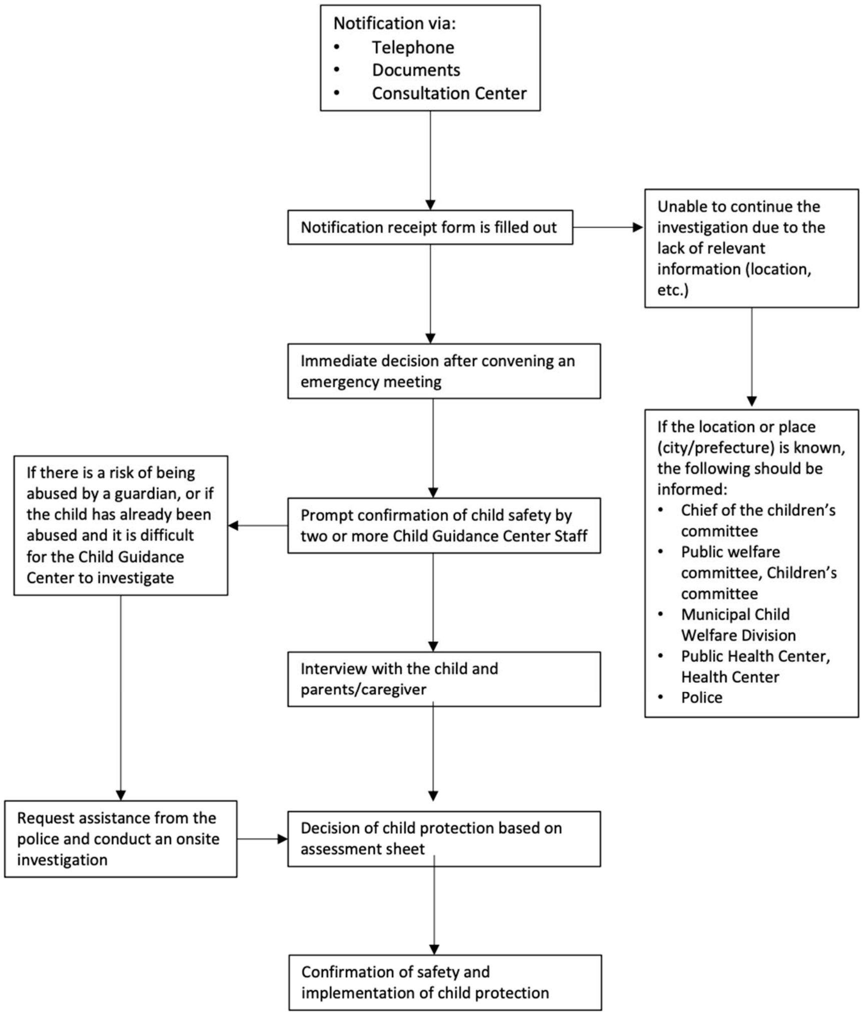

(translated from MHLW [2])

Data management

Monthly number of child abuse cases per prefecture were transformed to monthly child abuse rates using the prefecture-specific under-18 population of 2020 obtained from the Vital Statistics [3]; as shown in Supplementary Equation 1 (SEq1).

$$\widehat{Y_{c,t}} = \frac{Y_{c,t}}{pop_c} \times 100{,}000\ population \qquad \text{[SEq1]}$$

Whereby $Y_{c,t}$ is the monthly number of child abuse consultations for time *t*, prefecture *c*. pop_c is the 2020 prefecture-specific under-18 population. $\widehat{Y_{c,t}}$ is the child abuse consultation rate.

Data analysis

© 2023 Seposo X et al. *JAMA Network Open.*

We parameterized the first-stage, prefecture-level association using SEq2 (below). Here, we employed a classical single-series, interrupted time series design.

$$\widehat{Y_{c,t}} \sim Quasipoisson$$
$$\widehat{Y_{c,t}} = \beta_1 time + \beta_2 covid + \beta_3 timelapse + factor(month) + \varepsilon \qquad [SEq2]$$

$\widehat{Y_{c,t}}$ is the child abuse consultation rate per prefecture, per unit of time, which was assumed to follow a Poisson distribution accounting for overdispersion via a quasi-likelihood estimation, simply Quasi-Poisson. *time* is the temporal variable representing the time since the start of the study period. *covid* is a binary variable representing the pre-pandemic period (coded as 0) or the pandemic period (coded as 1). *timelapse* is the time since the start of the occurrence of the event; in this case the pandemic. Owing to the potential seasonal trend in the child abuse consultation rates (Figure 1, lower panel), *month* is treated as a categorical variable to adjust for the potential seasonal pattern of the child abuse consultations. ε is the error term. β_1, β_2 and β_3 are the coefficients of association representing the change in the outcome variable associated with unit increase in time (or the pre-pandemic trend), level change, and slope change, respectively.

In this study, first-stage, prefecture-specific coefficients (β_3) were then pooled using a univariate random-effects meta-analysis to generate the nationwide effects estimates of the difference between the slope of pre-pandemic and pandemic periods.

Sensitivity analyses

We ran the analysis restricting the pandemic period to 2020 only (*results not shown*). The current study, which compared 2019 versus 2020-2021 periods, has a pooled Odds Risk (OR) of 0.92 (95% Confidence Interval (CI): 0.87, 0.97), whereas 2019 versus 2020 only had a pooled OR of 0.85 (95% CI: 0.79, 0.91). We did not find any statistical difference (P = 0.10) between the two ORs, thus we retained the current 2019 versus 2020-2021 analysis.

eReferences

1. MHLW. Child abuse prevention measures. 2022. https://www.mhlw.go.jp/stf/seisakunitsuite/bunya/kodomo/kodomo_kosodate/dv/index.html
2. MHLW. A Guide to Responding to Child Abuse. MHLW. 2022.
3. MIAC. eStat. 2022. https://www.e-stat.go.jp/en/about

© 2023 Seposo X et al. *JAMA Network Open.*
