# Supplementary material for: Child Abuse Consultation Rates Before vs During the COVID-19 Pandemic in Japan
Source: JAMA Netw Open. 2023 Mar 9;6(3):e231878. doi: 10.1001/jamanetworkopen.2023.1878 (PMC9999241; doi:10.1001/jamanetworkopen.2023.1878)
Supplement: Supplement 2. — Data Sharing Statement [file jamanetwopen-e231878-s002.pdf]

Data Sharing Statement

Seposo. Child Abuse Consultation Rates Before vs During the COVID-19 Pandemic in Japan. *JAMA Netw Open*. Published March 09, 2023. doi:10.1001/jamanetworkopen.2023.1878

Data
Data available: Yes
Data types: Data (not involving human participants)
How to access data: This can be access via appropriate request from the corresponding author; Xerxes T. Seposo.
When available: With publication

Supporting Documents
Document types: None

Additional Information
Who can access the data: This can be access via appropriate request from the corresponding author; Xerxes T. Seposo.
Types of analyses: Secondary analyses.
Mechanisms of data availability: This can be access via appropriate request from the corresponding author; Xerxes T. Seposo.
